# Functional and Evolutionary Significance of Human MicroRNA Seed Region Mutations

**DOI:** 10.1371/journal.pone.0115241

**Published:** 2014-12-12

**Authors:** Christopher G. Hill, Neda Jabbari, Lilya V. Matyunina, John F. McDonald

**Affiliations:** Integrated Cancer Research Center, School of Biology and Parker H. Petit Institute of Bioengineering and Biosciences, Georgia Institute of Technology, 315 Ferst Dr., Atlanta, Georgia 30332-0363, United States of America; The John Curtin School of Medical Research, Australia

## Abstract

MicroRNAs have emerged in recent years as important regulators of cell function in both normal and diseased cells. MiRNAs coordinately regulate large suites of target genes by mRNA degradation and/or translational inhibition. The mRNA target specificities of miRNAs in animals are primarily encoded within a 7 nt “seed region” mapping to positions 2–8 at the molecule's 5′ end. We here combine computational analyses with experimental studies to explore the functional significance of sequence variation within the seed region of human miRNAs. The results indicate that a substitution of even a single nucleotide within the seed region changes the spectrum of mRNA targets by >50%. The high functional cost of even single nucleotide changes within seed regions is consistent with their high sequence conservation among miRNA families both within and between species and suggests processes that may underlie the evolution of miRNA regulatory control.

## Introduction

MicroRNAs (miRNAs) are small 20–22 nucleotide (nt) RNA molecules that play important regulatory roles in cell function [1], embryonic development [2] and the onset and progression of a variety of diseases [3], including cancer [4]. Like siRNAs and other small regulatory RNAs, miRNAs regulate their target genes by mRNA degradation and/or translational inhibition [5]. However, unlike siRNAs that target one or a few genes, individual miRNAs have evolved the ability to coordinately regulate large suites of target genes, many of which may encode coordinated cellular functions [5], [6]. The mRNA target specificities of miRNAs in animals are primarily encoded within a 7 nt “seed region” mapping to positions 2–8 at the molecule's 5′ end [7], [8]. The importance of this 7 nt sequence to miRNA function is evidenced by the fact that the seed region sequence of many miRNA families is highly conserved both within and between species [9]. Mature single-stranded miRNAs bound to the RNA-induced silencing complex (RISC) recognize their regulatory targets by Watson-Crick base pairing to compatible sequences (usually in 3′ un-translated regions or 3′ UTRs) in target mRNAs.

It is estimated that there are >1000 sequentially distinct miRNAs in the human genome, each being present in a few to hundreds of copies [10]. We focused on 249 human miRNAs previously shown to be sequentially conserved across mammalian species [11]. In this study, we combine computational analyses with experimental studies to explore the functional significance of sequence variation within the seed region of human miRNAs. Our computational analyses predict that as few as one nucleotide change within this 7-nt seed region will alter the spectrum of targeted mRNAs by >60–70%. Further nucleotide substitutions are predicted to have little to no additional effect. Ectopic over expression of synthetic miRNAs with variable seed regions (miR-429, miR-141 and miR-205) but with identical (miR-429) non-seed regions were conducted to experimentally evaluate the consequence of differences in seed region on patterns of gene expression. The experimental results again indicate that as few as one nucleotide change within seed regions results in >50% alteration in the spectrum of mRNAs directly or indirectly regulated by the over expressed miRNA. Further nucleotide differences (5 nucleotide differences) within the seed region were found to have no additional effect. The high functional cost of even a single nucleotide change within the seed region of human miRNAs is consistent with the rigidly conserved seed sequence identity among miRNA families both within and between species [9] and suggests possible mechanisms underlying the evolution of miRNA regulatory control.

## Materials and Methods

### Computational predictions of miRNA target and overlap

To determine the mRNA targets of the 249 conserved miRNAs, we utilized three online target prediction programs, miRanda-mirSVR [12], TargetScan [13] and PicTar [14]. The miRanda predictions are driven by mirSVR, an application that uses machine learning to evaluate and score the importance of various features from miRNAs and their putative target sites. Predicted miRNA targets were filtered for targets with a mirSVR score less than −0.2 to minimize false positives. Corroborative predictions were carried out using TargetScan and PicTar.

We determined the distance between two sequences by calculating the Hamming distance [15]. That distance is calculated by counting the number of nucleotide changes needed to transform one seed sequence into another. Overlap between the predicted miRNA targets was determined using cosine similarity [16], calculated by dividing the total number of overlapping genes by the square root of the product of the number of genes targeted by each miRNA. Taking the square root of the number of predicted targets reduces the influence of miRNAs with abnormally large numbers of targets and simultaneously normalizes the result, generating a score between 0 and 1. The significance of the difference in overlap of differentially expressed (DE) genes between two pairs of miRNAs was calculated using the chi-square test of association; the overlap and cost (non-overlap) for each pair were compared.

### Cell culture and transfections

HEY [17] ovarian cancer cells, provided by Gordon B. Mills (MD Anderson Cancer Center, Houston, TX), were cultured in RPMI-1640 (Mediatech, Manassas, VA) with 10% Fetal Bovine Serum (FBS, Atlanta Biologicals, GA) and 1% antibiotic-antimycotic solution (Mediatech-Cellgro, Manassas, VA), and incubated at 37°C and 5% CO_2_. The transfection protocol was as described previously [18]. Briefly, triplicate wells of exponentially growing cells were transfected with miR-429, and the custom designed miRNAs M12, M14 and M5 purchased as Pre-miR miRNA Precursors (Ambion, Austin, TX). Transfections were performed using Lipofectamine 2000 transfection reagent (Invitrogen, Carlsbad, CA) according to the manufacturer's instructions. Ambion Pre-miR miRNA Precursor Negative Control was used as a negative control.

### RNA isolation and whole genome microarray

RNA was extracted from transfected cells using the RNeasy Mini RNA isolation kit (QIAGEN, Valencia, CA). Microarray experiments were performed as previously described [19]. Briefly, RNA samples with high integrity were converted to cDNA and amplified with Applause 3′-Amp System (NuGen, San Carlos, CA). The cDNA was fragmented and Biotin labeled using the Encode Biotin Module (NuGen). Labeled cDNA was then hybridized to Affymetrix HG-U133 Plus 2.0 arrays and analyzed with GeneChip Scanner 3000 (Affymetrix, Santa Clara, CA).

### Microarray data analysis

To determine differentially expressed genes in triplicates of experimental miRNA and negative control treated cells, the following procedure was followed. Quality control was first assessed using all raw CEL files as implemented in Array Analysis [20]. GC Robust Multi-array Average (GCRMA) normalization was performed using all CEL files that passed quality control (n = 3 per experimental group after quality assessment). For GCRMA normalization, each experimental miRNA group was compared independently to the negative control group. Next, probe set filtering was performed as follows: present/absent calls were first generated by Microarray Suite 5.0 (MAS5.0) using the Affymetrix Expression Console v1.1. Based on present/absent calls, probe sets with less than 50% present calls across all experimental miRNA and negative control samples were removed. In addition, probes and probe sets lacking a gene symbol annotation were ignored. Based on GCRMA expression signals, calculation of signal-to-noise ratio (SNR  =  mean/standard deviation) was used to select and keep only the probe sets with the highest SNR for differential expression profiling. GCRMA expression signals for those probe sets were submitted to SAM (Significance Analysis of Microarrays) [21]. Each SAM input file contained triplicates of experimental miRNA and negative control GCRMA expression columns. Differentially expressed genes in each experimental miRNA group were determined compared to the negative control (as a baseline) using the false discovery rate (FDR) <2%. SAM output files included significantly differentially expressed genes (upregulated and down-regulated genes in separate lists). All raw CEL files, MAS5.0 (CHP files) and GCRMA processed files are deposited in the Gene Expression Omnibus (http://www.ncbi.nlm.nih.gov) SuperSeries number GSE56973.

### Orthologous genes

Orthologous genes in human and mouse were identified using the BioMart data-mining tool [22]. Briefly, all *Homo sapiens* genes were selected on BioMart and filtered to remove the genes with no mouse orthologs. The output file included the mouse orthologs for all the resulting genes. These genes were then used for our comparison of miR-429 and miR-200b predictions across human and mouse species.

## Results and Discussion

**Figure 1:**
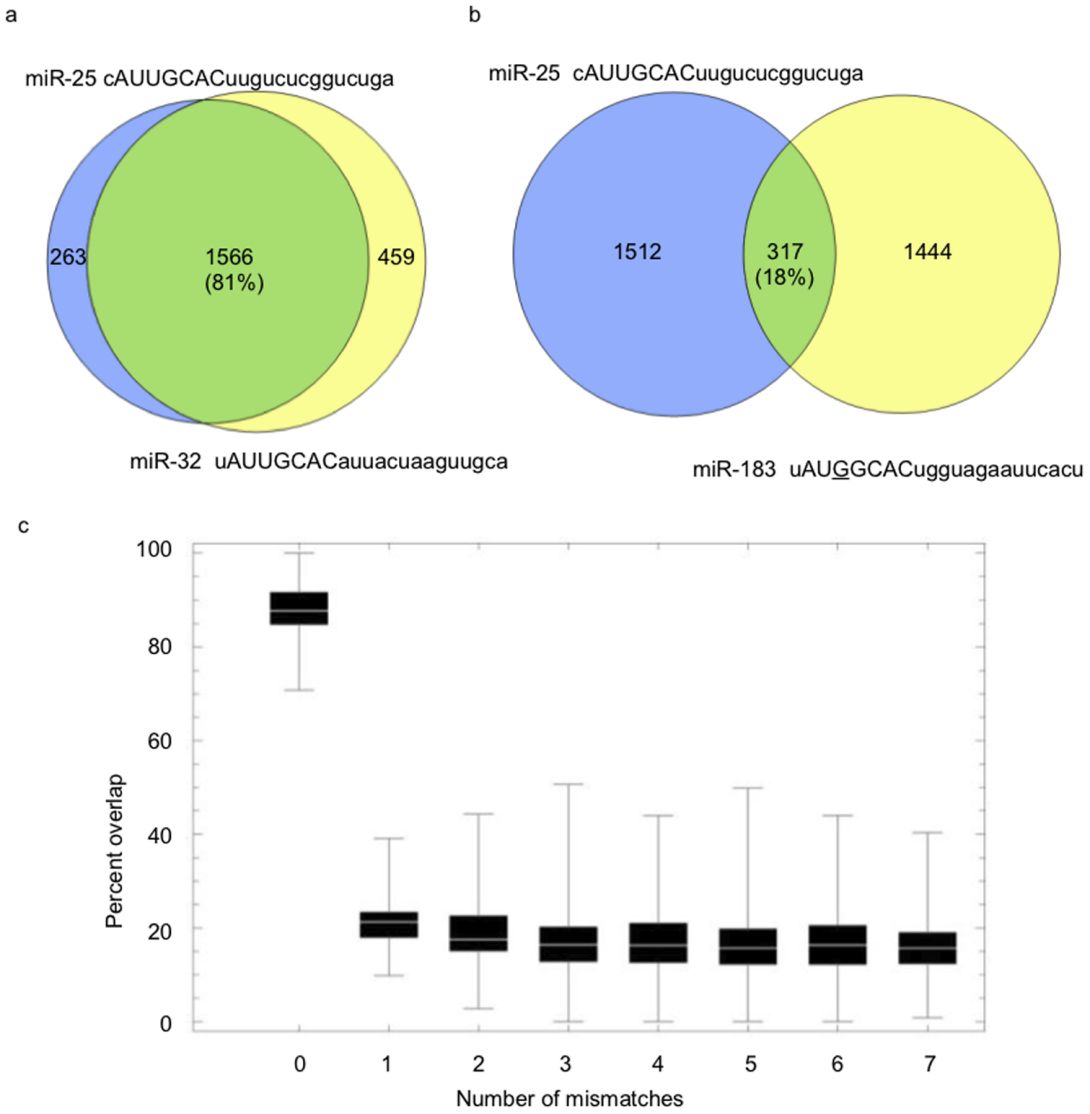
A single nucleotide difference in seed region of miRNAs is associated with a major change in regulated target genes. A) miR-25 and miR-32, two miRNAs with identical seed regions (upper-case letters), have 81% overlap in their predicted target genes; B) miR-25 and miR-183, two miRNAs with a single nucleotide difference within their respective seed regions have only 18% overlap in their predicted target genes; C) The overlap of predicted targets for all 249 pairs of conserved miRNAs grouped by the number of mismatches in their respective seed regions. The computed percentage overlaps (cosine similarity) are presented as box and whisker plots [the bottom and top of each box represent the first and third quartiles of variation while the band inside the box represents the median (second quartile) value; the “whiskers” represent variability outside the upper and lower quartiles].

The functional consequence or “cost” of seed region nucleotide changes involves the loss of regulatory control over previously targeted mRNAs and/or the acquisition of novel regulatory control over previously untargeted mRNAs. To systematically explore this phenomenon computationally, we first determined the number of nucleotide changes needed to transform one seed region into another (Hamming distance) for each of the 249 miRNAs analyzed in this study. We then calculated the percent overlap (cosine similarity) of predicted targets for all pairs of miRNAs having identical seeds. For example, the percent target gene overlap for miR-25 and miR-32 (both having the seed sequence: 5′AUUGCAC) predicted by miRanda-mirSVR is 81% (Fig. 1A). The 19% (100%–81%) divergence in non-overlapping targets is attributable to sequence variation mapping to the non-seed regions (see S1a Figure for an additional example). The median percent overlap between all pairs of conserved miRNAs with identical seeds is 88% (Fig. 1C; S1 Table; n = 144), with an average of 12% divergence in non-overlapping genes attributable to variation in non-seed regions.

We next independently computed the average percent overlap of predicted mRNA targets for pairs of miRNAs having seeds that differ by 1 to 7 nucleotides. The results (Figs. 1B, 1C, S1B; S2 Table) indicate that even a single nucleotide mismatch in the seed regions of two miRNAs is computationally predicted to reduce the percent overlap (and increase the percent of non-overlap) among their respective targeted mRNAs by >70%. The generality of these computational predictions was corroborated independently by conducting the same analyses using two additional target prediction algorithms, TargetScan and PicTar (S2A, S2B Figures). Thus, the computational studies consistently predict that as few as one nucleotide substitution within the seed region of miRNAs will be associated with significant functional cost and that further changes will have little or no additional cost.

**Figure 2:**
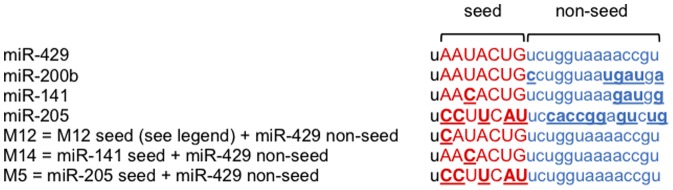
Sequence alignments of miR-429, miR-200b, miR-141, miR-205, M12, M14 and M5. Seed and non-seed regions are colored in red and blue, respectively. Differences in seed and non-seed regions of miRNAs relative to miR-429 are underlined. M12, 14 and M5 all have identical non-seed regions with miR-429. M12 differs in seed region sequence from miR-429 by one nt at position 2. The seed region sequence of M14 is identical to that of miR-141 and differs from the seed of miR-429 by one nucleotide at position 4. M5 differs in seed region sequence from miR-429 by 5 nucleotides.

While informative in their own right, functional predictions based on target prediction algorithms alone are often inaccurate *in vivo* because they ignore the myriad of indirect regulatory effects induced by miRNAs [6], [23], [24]. In an effort to experimentally explore the functional cost of nucleotide variation within miRNA seed regions, we selected for analysis members of the miR-200 family of human miRNAs that differ by a single nucleotide in the seed region (miR-429 vs. miR-141 differ by 1 nucleotide at position 4) or differ by multiple nucleotides (miR-429 and miR-205 differ by 5 nucleotides at positions 2,3,5,7 and 8) within their respective seed regions (Fig. 2). In order to avoid confounding effects attributable to variation within non-seed regions and to focus on the significance of seed region variation, synthetic derivatives of these naturally occurring miRNAs were constructed to have identical miR-429 non-seed regions (Fig. 2). In addition, to explore the possible significance of variability in the position of single nucleotide differences within seed regions, we constructed an additional miR-429 variant with a single nucleotide substitution at seed region position 2 (M12, Fig. 2). Each of these miRNAs were independently transfected into the well-characterized HEY ovarian cancer cell line [17] and after 48 hrs RNA was extracted and subjected to gene expression analysis (Affymetrix, U133) as previously described [18], [19]. (S3–S6 Tables for detailed results).

**Figure 3:**
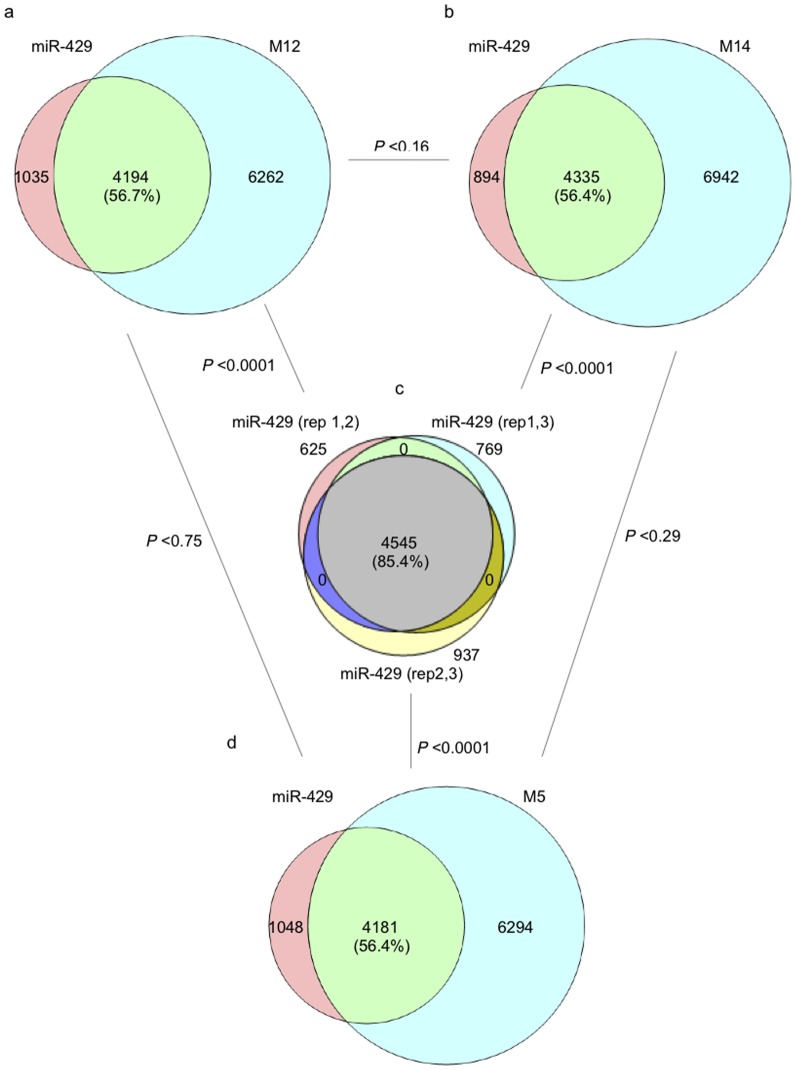
Overlap among differentially expressed (DE) genes in HEY cells after ectopic expression of miRNAs with different seed region sequences. An overlap of 85% in differentially expressed genes was observed among 3 replicate experiments in which miR-429 was ectopically expressed in HEY cells (C). A single nucleotide change in the seed region of miR-429 (M12, M14) resulted is a significant reduction (56% vs 85%) in the overlap among differentially expressed genes (A, B). Differences in the position of this single nucleotide difference (M12, position 2 vs M14, position 4) did not have a significant effect on degree of overlap (A vs. B). Five nucleotide differences in the seed region of miR-429 (M5) also resulted in a significant reduction in overlap (56% vs. 85%) among differentially expressed genes (D vs. C) but not significantly different from miRNAs differing by only 1 nucleotide difference from miR-429 (D vs. A; D vs B).

As mentioned above, the functional cost associated with nucleotide changes within seed regions is reflective of the loss of regulatory control over previously targeted mRNAs and/or the acquisition of novel regulatory control over previously untargeted mRNAs. We experimentally estimated these parameters by comparing all significantly differentially expressed genes in cells transfected by miRNAs with seeds differing by 0 (miR-429 vs miR-429), 1 (miR-429 vs M12 and miR-429 vs M14) or 5 (miR-429 vs M5) nucleotides. Presented in Fig. 3C is the observed percent overlap of all significantly differentially expressed genes among 3 replicate transfections with miR-429. The high similarity in percent overlap among these replicate miR-429 transfections is indicative of the low experimental error associated with the technique. Figs. 3a and 3b present the observed percent overlap in significantly differentiated genes in cells transfected with miR-429 vs cells transfected by miRNAs differing from miR-429 by a single nucleotide within their respective seed regions. The differences are highly significant (χ^2^ = 1511; p<0.0001) and consistent with the prediction that a substantial functional cost is associated with even a single nucleotide change within miRNA seed regions. Differences in overlap between the miR-429 vs M12 and miR-429 vs M14 comparisons were not found to differ significantly (χ^2^ = 1.95; p<0.16) indicating that, in this experimental context, position of the single nucleotide change is not significant with respect to functional cost.

Changes in the percent overlap of significantly differentially expressed genes between cells transfected by miR-429 vs those transfected by the miRNA differing at 5 nucleotide positions within the seed region (Fig. 3D) were also highly significant (χ^2^ = 1535; p<0.0001) but not significantly different from the changes induced by miRNAs with only a single nucleotide difference from miR-429 (χ^2^ = 0.1; p<0.75).

**Figure 4:**
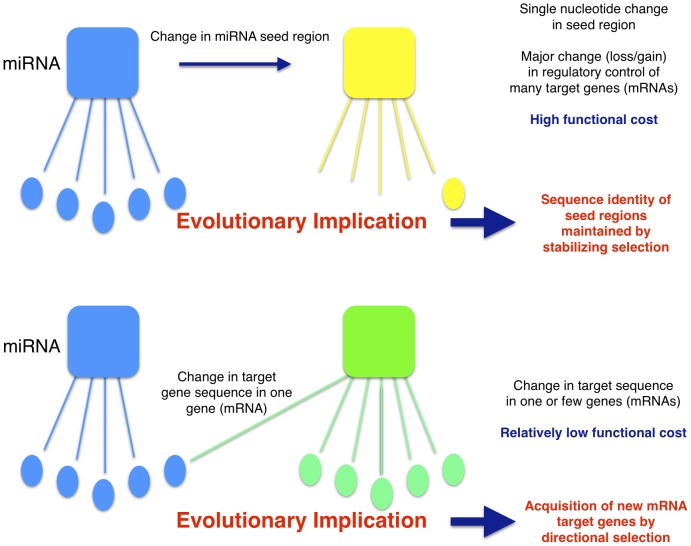
The high functional cost of even single nucleotide changes in miRNA seed regions implies a mechanism of miRNA regulatory evolution. If a miRNA (blue square) changes a single nt within its seed region (yellow square), a significant fraction of its target genes (blue and yellow ovals) will change resulting in high functional cost. As a result, strong stabilizing selection is expected to maintain miRNA seed region sequence identity among miRNA families within and between species. In contrast, individual genes may change miRNA regulatory control(s) (blue to green squares) by as little as a single nucleotide change in their target sequences (blue to gene ovals) with relatively low functional cost. Thus, evolutionary changes in miRNA regulatory control are likely to be driven primarily by random and/or adaptive (directional selection) changes in target gene sequences.

Collectively, the above results indicate that even a single nucleotide substitution within the seed regions of miRNAs is associated with substantial functional cost and suggests an evolutionary model whereby strong stabilizing selection is maintaining rigid conservation of miRNA seed sequences both within and between species. Individual target genes, on the other hand, may acquire and/or lose miRNA regulatory control(s) through even single nucleotide substitutions in miRNA target sequences complimentary to miRNA seeds (typically within 3′ UTRs) (Fig. 4). Any functional consequence of such mutations would be incurred on the individual gene level rather than on the multi-gene level associated with miRNA seed region mutations. This implies that although seed regions may be highly conserved both within and between species due to strong stabilizing selection, the spectrum of genes regulated by these sequentially conserved miRNAs may be expected, on average, to vary significantly, especially between more distantly related species where there has been ample time/opportunity for individual genes to acquire variation in their target sequence(s) and to re-associate themselves with other, presumably adaptive, miRNA regulatory controls (directional selection).

**Figure 5:**
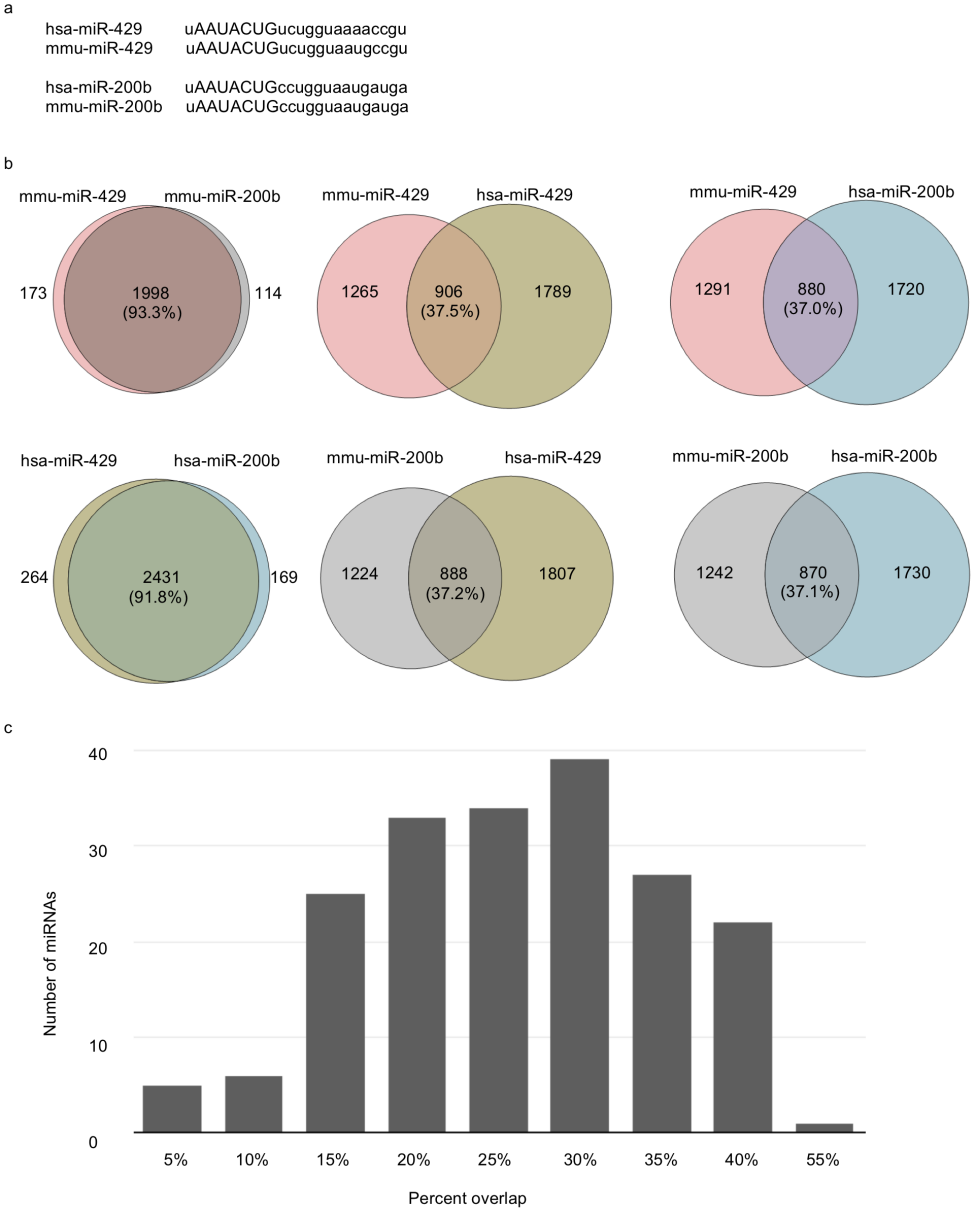
Mouse and human miRNAs share sequentially identical seed regions but regulate highly divergent groups of target genes. A) Sequence alignment between human (has-miR-429/200b) and mouse (mmu-miR-429/200b) miR-429 and miR-200b miRNAs. Despite the substantial evolutionary distances between these two species, the respective seed regions are sequentially identical indicative of strong stabilizing selection. B) Venn diagrams showing the % overlap in predicted miR-429 and miR-200b target genes between human and mouse orthologs. C) The average percent overlap of genes predicted to be targeted by all 249 sequentially conserved mouse and human miRNAs is <30%. The results are consistent with a model of miRNA regulatory evolution whereby miRNA seed region sequences are selectively conserved while target genes may rapidly re-position themselves under novel miRNA regulatory control(s).

As an initial test of this prediction, we selected two miRNAs (miR-429 and miR-200b) that have identical seed regions in both humans and mice (Fig. 5A). We employed the miRanda-mirSVR algorithm to predict the respective orthologous mRNA targets of these two miRNAs (human: hsa-miR-429, hsa-miR-200b; mouse: mmu-miR-429, mmu-miR-200b) in both species. As shown in Fig. 5B, the percent overlap between the predicted gene targets of these two miRNAs (intra-specific) is >90% (mouse: 93.3%; humans: 91.8%) in both species. However, despite the fact that the human and mouse miRNAs share sequentially identical seed regions, they display <40% overlap among their respective target genes/mRNAs in the non-native species (inter-specific) (Fig. 5B). To determine if these differences are representative of other sequentially conserved miRNAs, we computed the percent overlap of genes targeted by the 249 miRNAs sequentially conserved in mouse and humans. The results confirm that the average overlap between targeted genes in mouse and humans is <30% (Fig. 5C). This dichotomy is well below the false positive values expected given the high stringency cut-off values used in our predictions (mirSVR score <−0.2) [12]. These results are consistent with the hypothesis that while miRNA seed regions may be selectively conserved across species, target genes maintain relative flexibility to acquire and/or lose miRNA regulatory controls by even single nucleotide changes within their respective miRNA target sequences (typically within 3′ UTRs).

Collectively, our findings support an evolutionary model whereby miRNAs initially evolve to regulate large suites of target genes. Thereafter, the sequential integrity of miRNA seed regions is maintained by strong stabilizing selection due to the high functional cost of even a single nucleotide mutation within miRNAs. In contrast, nucleotide mutations in the target sequences of individual genes, being, on average, of substantially lower functional cost, allow for a relatively rapid repositioning of miRNA-target gene associations. Indeed, a variety of scenarios might arise to buffer the possible negative effects of target sequence mutations in regulated genes. For example, duplication of specific target sequences within regulated genes could serve to mask the impact of the sudden loss of existing miRNA regulatory controls while still permitting genes to explore the potential adaptive benefits of acquiring new miRNA regulatory controls.

## Supporting Information

S1 Figure **One nucleotide difference or 7 nucleotide differences in the seed regions of miRNAs is predicted to be associated with equivalent levels of change in regulated target genes.** (**a**) miRNAs with identical seeds, miR-195 and miR-16, are predicted to share 91% of their target genes; (b) miRNAs with a single nucleotide change in their seed regions, miR-195 and miR-29a, are predicted to share only 21% of their target genes; (c) miRNAs with no sequence similarity in their seed regions, miR-195 and miR-205, are predicted to share only 18% of their target genes. These results are consistent with the hypothesis that as little as a single nucleotide difference within miRNA seed regions has a major effect on miRNA regulatory control.
(PDF)

S2 Figure **Distribution of percent overlap of miRanda-mirSVR predicted targets of miRNAs having 0 through 7 seed mismatches.** Insets show distribution according to two other popular prediction algorithms: a) TargetScan, and b) PicTar. The results are uniformly consistent with the prediction that even a single nucleotide mismatch within miRNA seed regions results in a large change in targeted mRNAs.
(PDF)

S1 Table **Target overlap for miRNAs with identical seeds.** Predicted (miRanda-mirSVR) overlap (cosine similarity) in target genes for all pairs of miRNAs with identical seed regions.
(XLSX)

S2 Table **Target overlap for miRNAs with non-identical seeds.** Predicted (miRanda-mirSVR) overlap (cosine similarity) in target genes for all pairs of miRNAs with non-identical seed regions.
(XLSX)

S3 Table **Fold-change for all (5229) significantly differentially expressed genes after ectopic expression of miR-429 in HEY cells.**
(XLS)

S4 Table **Fold-change for all (10,456) significantly differentially expressed genes after ectopic expression of M12 in HEY cells.**
(XLS)

S5 Table **Fold-change for all (11,277) significantly differentially expressed genes after ectopic expression of M14 in HEY cells.**
(XLS)

S6 Table **Fold-change for all (10,475) significantly differentially expressed genes after ectopic expression of M5 in HEY cells.**
(XLS)
