# Supplementary material for: Functional and Evolutionary Significance of Human MicroRNA Seed Region Mutations
Source: PLoS One. 2014 Dec 12;9(12):e115241. doi: 10.1371/journal.pone.0115241 (PMC4264867; doi:10.1371/journal.pone.0115241)
Supplement: S1 Figure — One nucleotide difference or 7 nucleotide differences in the seed regions of miRNAs is predicted to be associated with equivalent levels of change in regulated target genes. (a) miRNAs with identical seeds, miR-195 and miR-16, are predicted to share 91% of their target genes; (b) miRNAs with a single nucleotide change in their seed regions, miR-195 and miR-29a, are predicted to share only 21% of their target genes; (c) miRNAs with no sequence similarity in their seed regions, miR-195 and miR-205, are predicted to share only 18% of their target genes. These results are consistent with the hypothesis that as little as a single nucleotide difference within miRNA seed regions has a major effect on miRNA regulatory control. (PDF) [file pone.0115241.s001.pdf]

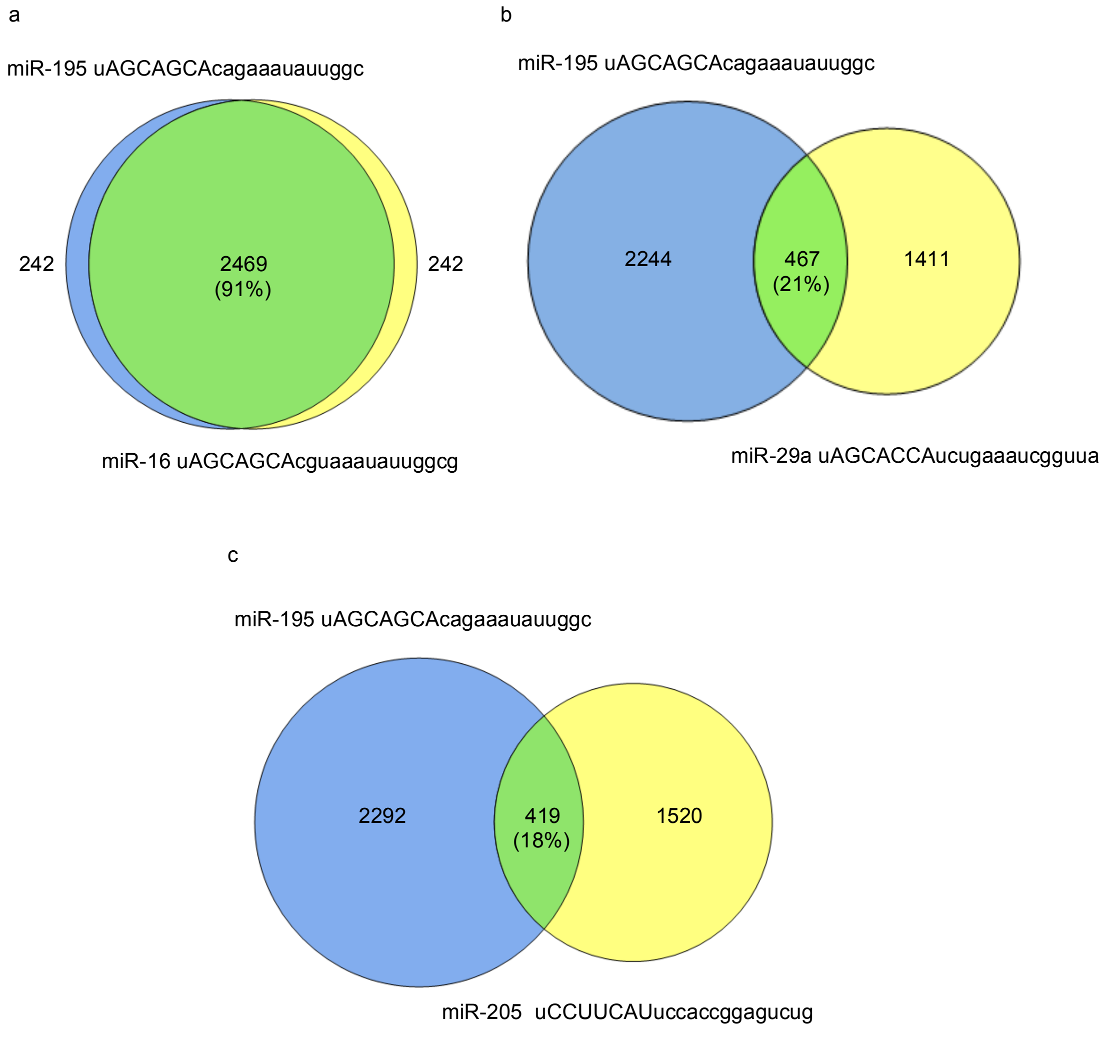
a
miR-195 uAGCAGCAcagaaauauuggc
242
2469
(91%)
242
miR-16 uAGCAGCAcguaaauauuggcg
b
miR-195 uAGCAGCAcagaaauauuggc
2244
467
(21%)
1411
miR-29a uAGCACCAucugaaaucgguua
c
miR-195 uAGCAGCAcagaaauauuggc
2292
419
(18%)
1520
miR-205 uCCUUCAUuccaccggagucug
