# Supplementary figures and images for: Functional and Evolutionary Significance of Human MicroRNA Seed Region Mutations
Source: PLoS One. 2014 Dec 12;9(12):e115241. doi: 10.1371/journal.pone.0115241 (PMC4264867; doi:10.1371/journal.pone.0115241)

Percent overlap

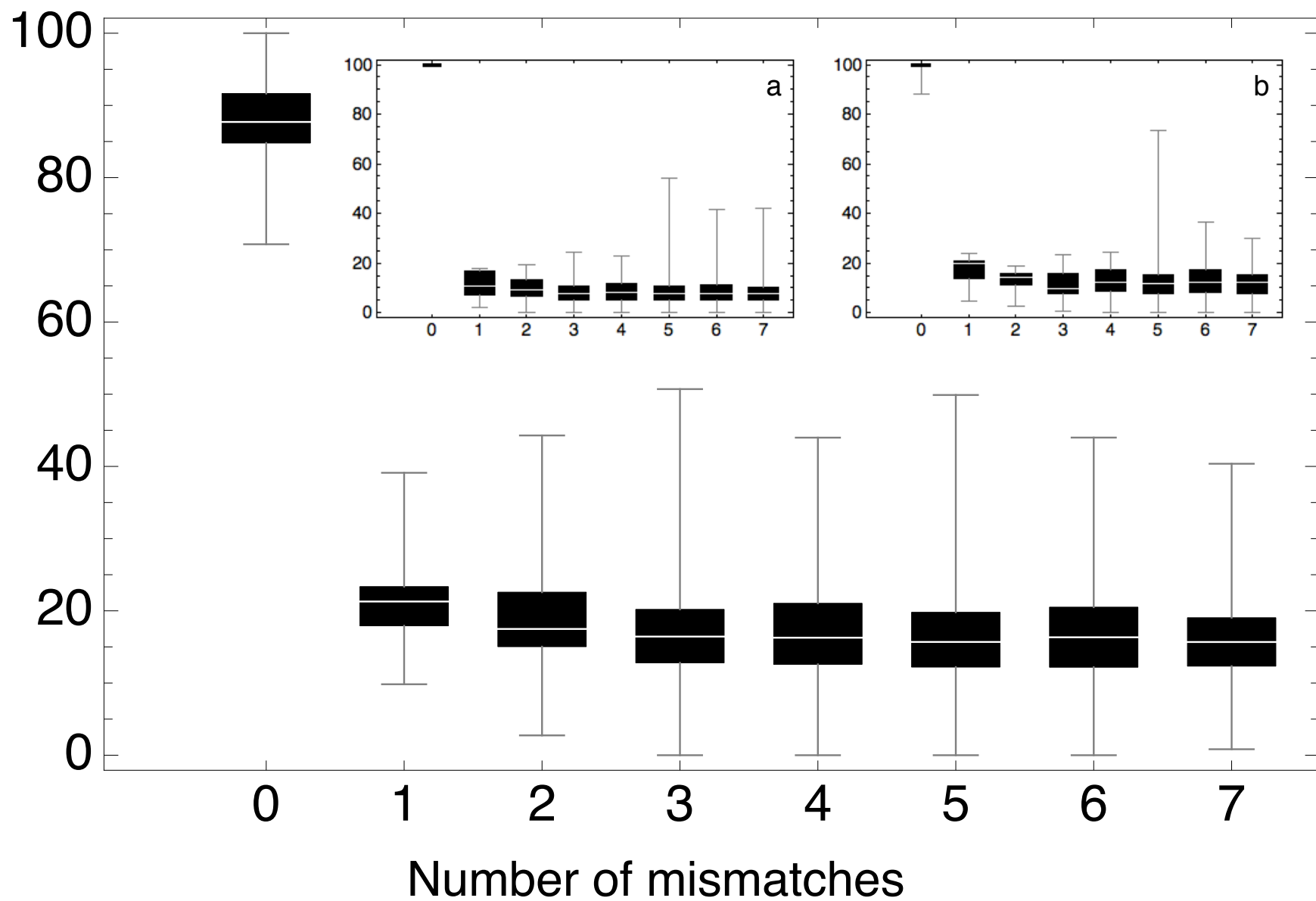

Supplement: S2 Figure — Distribution of percent overlap of miRanda-mirSVR predicted targets of miRNAs having 0 through 7 seed mismatches. Insets show distribution according to two other popular prediction algorithms: a) TargetScan, and b) PicTar. The results are uniformly consistent with the prediction that even a single nucleotide mismatch within miRNA seed regions results in a large change in targeted mRNAs. (PDF) [file pone.0115241.s002.pdf]
